# 4402 The effects of hemodilution *in vitro* on coagulation in term parturients using thromboelastometry

**DOI:** 10.1017/cts.2020.434

**Published:** 2020-07-29

**Authors:** Chloe Getrajdman, Matthew Sison, Hung-Mo Lin, Daniel Katz

**Affiliations:** 1 Icahn School of Medicine at Mount Sinai

## Abstract

OBJECTIVES/GOALS: Little is known about the effect of hemodilution with crystalloid on blood coagulation in obstetric patients. The purpose of our study was to examine the impact of hemodilution on components of blood coagulation using rotational thromboelastometry (ROTEM®) in term parturients METHODS/STUDY POPULATION: This is a prospective, observational pilot study including 35 healthy, pregnant patients at term (≥37 weeks) without history of bleeding or clotting disorder or on medication affecting coagulation. Venous blood samples were collected from all patients and divided into specimen tubes to generate varying degrees of hemodilution with Plasma-Lyte (0%, 20%, 25%, 30%, 35%, 40%, 45%, 55%, 60%, 65%, 70%, 75%, 80%). Rotational thromboelastometry was then performed on samples to assess for coagulation changes. RESULTS/ANTICIPATED RESULTS: EXTEM (extrinsically activated assay) clotting time (CT) became prolonged at 65% hemodilution and above, and the median CT was in the coagulopathic range (>80 seconds) at a dilution of 80%. FIBTEM (extrinsically activated assay with platelet inhibitor, primarily measuring contribution of fibrinogen to coagulation) amplitude at 5 minutes (A5) began to diminish at 35% hemodilution, with the median A5 in the coagulopathic range (<12 mm) at 55% hemodilution. The area under the curve (AUC), a marker of clot strength, for EXTEM and FIBTEM consistently declined as hemodilution increased. Greater decreases in FIBTEM AUC were seen compared to EXTEM AUC, with the ratio of FIBTEM:EXTEM AUC at each dilution demonstrating a statistically significant difference from baseline. DISCUSSION/SIGNIFICANCE OF IMPACT: All thromboelastometry values demonstrated a hypocoagulable trend as hemodilution increased. However, the samples analyzed by the FIBTEM assay trended toward a coagulopathy at a lower degree of hemodilution compared to the EXTEM assay. As FIBTEM tests analyze the role of fibrinogen in hemostasis and EXTEM tests analyze the role of platelets, our findings suggest that platelets may be able to withstand higher degrees of hemodilution before impairing hemostasis compared to fibrinogen. These findings support the growing body of literature that in early stages of severe obstetric hemorrhage, the prioritization of fibrinogen replacement may be critical in preventing further coagulopathy. CONFLICT OF INTEREST DESCRIPTION: All authors have no conflicts of interest to report.

